# Large-scale production and evaluation of marker-free *indica* rice IR64 expressing phytoferritin genes

**DOI:** 10.1007/s11032-013-9931-z

**Published:** 2013-08-11

**Authors:** Norman Oliva, Prabhjit Chadha-Mohanty, Susanna Poletti, Editha Abrigo, Genelou Atienza, Lina Torrizo, Ruby Garcia, Conrado Dueñas, Mar Aristeo Poncio, Jeanette Balindong, Marina Manzanilla, Florencia Montecillo, Maricris Zaidem, Gerard Barry, Philippe Hervé, Huxia Shou, Inez H. Slamet-Loedin

**Affiliations:** 1 Plant Breeding, Genetics, and Biotechnology Division, International Rice Research Institute, DAPO Box 7777, Metro Manila, Philippines; 2 State Key Laboratory of Plant Physiology and Biochemistry, College of Life Sciences, Zhejiang University, Hangzhou, China; 3 Bayer Cropscience NV, Technologie Park 38, 9052 Ghent, Belgium; 4 Max Planck Institute for Developmental Biology, Tuebingen, Germany

**Keywords:** Soybean ferritin, Rice ferritin, Transgenic rice, Agronomic evaluation, Marker-free

## Abstract

Biofortification of rice (*Oryza sativa* L.) using a transgenic approach to increase the amount of iron in the grain is proposed as a low-cost, reliable, and sustainable solution to help developing countries combat anemia. In this study, we generated and evaluated a large number of rice or soybean ferritin over-accumulators in rice mega-variety IR64, including marker-free events, by introducing soybean or rice ferritin genes into the endosperm for product development. Accumulation of the protein was confirmed by ELISA, in situ immunological detection, and Western blotting. As much as a 37- and 19-fold increase in the expression of ferritin gene in single and co-transformed plants, respectively, and a 3.4-fold increase in Fe content in the grain over the IR64 wild type was achieved using this approach. Agronomic characteristics of a total of 1,860 progenies from 58 IR64 single independent transgenic events and 768 progenies from 27 marker-free transgenic events were evaluated and most trait characteristics did not show a penalty. Grain quality evaluation of high-Fe IR64 transgenic events showed quality similar to that of the wild-type IR64. To understand the effect of transgenes on iron homeostasis, transcript analysis was conducted on a subset of genes involved in iron uptake and loading. Gene expression of the exogenous ferritin gene in grain correlates with protein accumulation and iron concentration. The expression of *NAS2* and *NAS3* metal transporters increased during the grain milky stage.

**Electronic supplementary material:**

The online version of this article (doi:10.1007/s11032-013-9931-z) contains supplementary material, which is available to authorized users.

## Introduction

Deficiency of iron (Fe) affects about 40 % of the global population (World Bank 2006). Deficiency in dietary Fe is the principal cause of anemia, affecting more than 2 billion people worldwide, with women and children most at risk. Iron deficiency can increase the chances of maternal and child mortality due to severe anemia, and can have negative consequences for cognitive and physical development of children, and for physical performance (World Health Organization 2001, 2008). Combating micronutrient malnutrition is considered to be among the best investments to generate a high return in socioeconomic benefits (The Copenhagen Consensus 2004; www.copenhagenconsensus.com/).

Rice (*Oryza sativa* L.) is the most important staple food crop in the world, and it constitutes as high as 75 % of the daily calorie intake (Khush 2005). Unfortunately, rice grain contains low Fe and zinc (Zn) (Ghandilyan et al. 2006). Biofortification to increase Fe content in rice endosperm could provide a low-cost, sustainable strategy to remedy Fe deficiency in populations consuming polished rice as a staple food (Bajaj and Mohanty 2005; Zhao and Shewry 2011).

One strategy to increase Fe in rice endosperm is by incorporating exogenous ferritin genes through transgenic technology. Ferritin is a class of iron storage protein that can store up to 4,500 atoms of Fe per molecule in its central cavity (Theil 1987; Briat and Lobréaux 1997). Plant ferritin subunit sequences share 39 and 49 % identity with mammalian ferritin sequences (Briat et al. 2010). Ferritin sequesters excess Fe and protects cells against Fe-toxic effects (Theil 1987).

A ferritin-bioengineered rice diet was demonstrated to be as effective as a FeSO_4_ diet in replenishing hematocrit, hemoglobin concentration, and liver Fe concentrations using a hemoglobin repletion assay in rats (Beard et al. 1996; Murray-Kolb et al. 2002). Similarly, Lönnerdal (2009) reported that soybean ferritin is absorbed comparably to FeSO_4_ by in vitro assessment using human intestinal (CaCo-2) cells and in vivo by using radiolabeled ferritin in human subjects. Ferritin is as bioavailable as FeSO_4_ in non-anemic women, based on the study of Davila-Hicks et al. (2004).

Increased iron concentration by incorporation of soybean ferritin genes has been reported in rice (Goto et al. 1999; Vasconcelos et al. 2003; Qu et al. 2005), with a maximum increase of 3.7-fold in seed Fe concentration. Paul et al. (2012) transformed using a rice ferritin gene, OsFer2, with modest increase (twofold) in Fe concentration. Johnson et al. (2011) obtained a 4.4-fold increase using a single transgene chelator approach, nicotianamine synthase (OsNAS). Masuda et al. (2012) and Wirth et al. (2009) successfully applied multigene approaches, combining ferritin with a Fe transporter and a chelator, both reporting a sixfold increase in Fe levels in the grain. Wirth et al. (2009) obtained a final concentration of 6 mg kg^−1^and Masuda et al. (2012) reported a maximum increase of up to 7 mg kg^−1^ in the greenhouse; however, seed Fe concentrations were reduced to 4 mg kg^−1^ under field conditions. In maize, Drakaki et al. (2005) combined two transgenes (soybean ferritin and *Aspergillus* phytase) to increase bioavailable Fe in the endosperm. None of the above rice biofortification studies have utilized a large number of events in an elite mega-variety and intensively evaluated different rice and soybean ferritins under field conditions. Incorporation of a nutritional trait into an elite mega-variety possessing agronomic and quality traits preferred by farmers would have a prompt and wider impact (Manzanilla et al. 2011).

The use of selectable markers is of great importance for the selection of transformed cells in which foreign DNA integration has taken place because of the low efficiency of transgene integration. Studies have shown that one of the common antibiotic resistance genes [the hygromycin phosphotransferase (*HPT*) gene] used in the present study has no biosafety concerns (European Food Safety Authority Scientific Panel 2004; Goldstein et al. 2005; CERA 2010). In recent years, for better public acceptance, different techniques have been employed to generate marker-free transgenic plants, including the co-transformation approach (Miki and McHugh 2004), site-specific recombination (Kondrák et al. (2006), homologous recombination (Hare and Chua 2002), and non-selected transformation. In the co-transformation approach, transformation is achieved using two separate plasmid vectors: one containing the gene of interest and the other the selective marker gene that targets insertion at two different loci in the plant genome, to be further eliminated by progeny segregation.

The objective of this study was to obtain high Fe concentration in polished rice that would be suitable for deregulation and adoption. A large number of transgenic plants containing the ferritin genes in the popular elite mega-variety IR64 was produced by the single gene approach in order to identify candidate events that have high Fe concentration in polished grain with good agronomic performance. This is the first study to perform extensive screening of a large population of progenies of transgenic rice events expressing the ferritin gene with and without the selectable marker under paddy soil conditions, suitable for easier deregulation as a potential transgenic product for rice farmers and consumers.

## Materials and methods

### Promoter selection

Three binary vectors were constructed to test the tissue-specific expression of rice endosperm-specific promoters, glutelin-1 (*GLUB1,* NCBI accession no. AY427569), glutelin-4 (*GLUB4,* NCBI accession no. AY427571), and globulin-1 (*GLB1,* NCBI accession no. AY427575), in IR64. The *GLUB1* promoter sequence corresponds to the –12 to –2,336 position of the promoter sequence, the *GLUB4* promoter sequence corresponds to the –12 to –1,474 position, and *GLB1* corresponds to the –1 to –840 position of the promoter sequence. The *GLUB1* promoter sequence was amplified using rice genomic DNA of Nipponbare using forward primer 5′-ACAGATTCTTGCTACCAACA-3′ and reverse primer 5′-ACGGATCCCCTTGCTTATGGAAACTTAAG-3′. The *GLUB4* promoter sequence was amplified using rice genomic DNA using forward primer 5′-TACAGGGTTCCTTGCGTGAA-3′ and reverse primer 5′-ACGGATCC ATGTTATTGGAAACTTGGGC-3′. *GLB1* was amplified using forward primer 5′-GTTAATCATGGTGTAGGCAA-3′ and reverse primer 5′-ACGGATCCGGTTGTTGTAGGACTAATGAAC-3′. The promoter fragments were cloned separately in pCR 2.1 TOPO (Invitrogen, San Diego, CA, USA), namely as pTOPO–*GLUB1*, pTOPO–*GLUB4* and pTOPO–*GLB1*.

These promoters were fused individually to the *GUSA* reporter gene in the binary vector pCAMBIA 1381Z (NCBI accession no. AF234306). The resulting vectors were transferred into *Agrobacterium* strain LBA4404 by the freeze–thaw method. These *Agrobacterium* strains harboring the different vectors individually were used to infect immature embryo from immature seeds of *Oryza sativa* cv IR64.

A histochemical β-glucuronidase (GUS) assay was performed as described by Jefferson et al. (1987) for dough stage [15 days after flowering (DAF)] and mature (30 DAF) seeds in selected transgenic lines and wild-type IR64. Reverse-transcriptase (RT) PCR using virA primers was performed to determine whether GUS expression due to *Agrobacterium* contamination was present in the tissue samples.

### Construct design of transformation vectors

Ferritin cDNAs were isolated from soybean (*Glycine max* L. cv PHI29924) and rice (*O. sativa* cv Nipponbare). Total mRNA was isolated from 1 g (fresh weight) of leaf tissues and the ferritin cDNAs *SoyFERH1* (0.914 kb), *SoyFERH2* (0.803 kb), *OsFER1C* (0.820 kb), and *OsFER2C* (0.821 kb) were amplified by RT-PCR using forward primer 5′-ACGTCGACCACAAATCTTAGCCGCCATT-3′ and reverse primer 5′-ACCTGCAGCCAGAATTTCAGAAAAGACCAAATG-3′ for *SoyFERH1*, and forward primer 5′-ACGTCGACTCGTTTTTCTTCCCAAATGG-3′ and reverse primer 5′-ACCTGCAGGGCCGTTCAAAGATTATACA-3′ for *SoyFERH2*. The *OsFER1C* cDNA sequence was amplified using forward primer 5′-TGCTGCAGCCTTTCCGCCATGCTTCCT-3′ and reverse primer 5′-TGACTAGTCCCATGGATGGAAGAAACGA-3′ while the *OsFER2C* cDNA sequence was amplified using forward primer 5′-TGCTGCAGATGCTTCCTCCTAGGGTTGC -3′ and reverse primer 5′-TGACTAGTCCATGGATGGAAGAAACGAA-3′. The ferritin cDNAs were then cloned separately in pCR 2.1 TOPO (Invitrogen), namely as pTOPO–*SoyFERH1,* pTOPO–*SoyFERH2,* pTOPO–*OsFER1C,* and pTOPO–*OsFER2C*. The cDNA clones were sent for sequencing (Macrogen, Korea) using universal primers M13 forward and M13 reverse. The sequences of the ferritin cDNA fragment were verified by alignment with known sequences of *SoyFERH1* (NCBI accession no. M64337), *SoyFERH2* (NCBI accession no. AB062754.1), *OsFER1C* (NCBI accession no. AF519570.1), and *OsFER2C* (NCBI accession no. AF519571.1).

Promoter cassettes were cut from pTOPO using restriction enzymes of *Eco*RI and *Bam*HI and cloned into the corresponding sites of pCAMBIA1380 with hygromycin phosphotransferase (HPT) selectable marker for one-vector transformation and pCAMBIA0380 without HPT selectable marker for two-vector co-transformation. Ferritin cDNAs that were first cloned into pTOPO vectors were then isolated using the restriction sites (*Sal*I and *Pst*I for *SoyFERH1* and *SoyFERH2*; *Pst*I and *Spe*I for *OsFER1C* and *OsFER2C*, respectively) and inserted into the corresponding enzyme sites downstream of the promoters.

### Production of transgenic plants with ferritin genes

The vectors were introduced into mega-variety IR64 by *Agrobacterium tumefaciens* strain LBA 4404. Rice immature embryos were co-cultivated with *Agrobacterium* following the modified procedure of Hiei and Komari (2006). For single transformation, the *Agrobacterium* strain used harbors a single transformation vector containing both the *HPT* and ferritin genes. For co-transformation to generate marker-free plants, a 5:1 mix ratio of *Agrobacterium* cultures of pCAMBIA 0380 containing the ferritin gene and pCAMBIA 1300 containing the *HPT* cassette was used.

### Molecular evaluation for the presence of transgenes

Transgenic rice lines were characterized for initial screening by PCR by using a primer set of the ferritin gene and *HPT* gene (forward primer 5′-TACTTCTACACAGCCATC-3′ and reverse primer 5′-TATGTCCTGCGGGTAAAT-3′). Positive plants were analyzed for the presence of a single-copy insert by DNA blotting. Rice genomic DNA was extracted from leaves using the procedure described by Dellaporta et al. (1983). Hybridization probes were chemically labeled with digoxigenin using a PCR DIG probe synthesis kit (Roche Diagnostic GmBH, Mannheim, Germany). Prehybridization, hybridization, and detection were carried out following the manufacturer’s instructions (Roche Diagnostic GmBH).

Plants with a single copy of the gene of interest and fertile plants were selected for biochemical and phenotypic evaluation.

### Phenotypic evaluation of transgenic plants

Phenotypes of the T1 and T2 single-copy events and their respective nulls were evaluated in the biosafety screenhouse. The marker-free plants were selected based on the presence of the ferritin gene and absence of *HPT*. T1 plants were transplanted following a randomized block design with three replicates. Each replicate of single events consists of seven PCR-positive and three null segregants and the wild type. Six parameters were recorded: (1) days to flowering, (2) plant height, (3) tiller number, (4) panicle number, (5) panicle length, and (6) panicle fertility. Days to flowering were counted from the start of sowing, while the length of the plant starting from above the soil to the tip of the tallest panicle was measured in centimeters for panicle length. For panicle fertility, all the panicles per plant were threshed; the filled and unfilled grains were separated using a grain blower and were counted afterwards. The data were analyzed statistically using Statistical Analysis Software (SAS) to determine whether there was a significant difference between the transgenic, null, and wild-type plants.

### Grain quality evaluation of high-Fe seeds

Seed samples of transgenic and non-transgenic plants were evaluated for the traits of amylose content, cooking quality, protein content, and milling potential score in IRRI’s Grain Quality and Nutrition Center Laboratory.

### Seed polishing method

Thirty brown rice grains were placed in 2-ml labeled micro tubes. The tubes were placed on a cryogenic metal rack (48 tubes per rack). The Genogrinder-2000 (SPEX CertiPrep Inc., Metuchen, NJ, USA) was set at 1,200 strokes min^−1^ for 2 min’ duration for 50 times. The samples were then placed in a new tube and run for an additional 20 times. Any trace of loose aleurone was removed.

### Homozygous T2 screening

Forty-five events were selected based on phenotypic performance and Fe concentration for T2 homozygous screening. Homozygous lines were determined based on a 90–100 % resistance response of 30 randomly selected seeds of individual lines in 40 mg/L hygromycin solution.

In addition, 20 plants of each T2 line were checked by PCR using the primers of target genes. The DNA sequences of the PCR primers used in this study are the following:

- *SoyFERH1*F: 5′-ATGGCTCTTGCTCCATCCAAAGTT-3′
- *SoyFERH1*R: 5′-TTGATCAAAGTGCCAAACACCGTG-3′
- *SoyFERH2*F: 5′-ACGTCGACTCGTTTTTCTTCCCAAATGG-3′
- *SoyFERH2*R: 5′-ACCTGCAGCGCCGTTCAAAGATTATACA-3′

### Iron concentration determination by ICP method

Polished seeds were sent to the Analytical Service Lab at IRRI for inductively coupled plasma–optical emission spectrometer (ICP-OES) analysis, and selected samples were validated by sending them to the Waite Analytical Laboratory, School of Agriculture, Food and Wine, University of Adelaide, Australia, for micronutrient concentration. Ten seeds (about 200 mg) from each plant were randomly selected, ground and wet-ashed with 2 ml HNO_3_ and H_2_O_2_ overnight at 110 °C. Ashing was repeated until the samples whitened. These samples were then dissolved in 15 ml of 1 N HCl. Concentration of Fe was measured using ICP-OES (Perkin Elmer ICP Optima 5300DV, Perkin Elmer, MA, USA) at 238.204 nm (Fe).

### ELISA, in situ Western, and Western blotting for ferritin protein expression

Total protein was extracted from transgenic plants and non-transgenic control plants. For ELISA, 96-well microtiter plates (Linbro, VA, USA) were coated with 100 μl dilution of purified crude protein (25 μg) from transgenic and control plants in sodium bicarbonate buffer (pH 8.6), and were incubated at 4 °C overnight. After blocking with 100 μl of blocking solution (10 mg ml^−1^ bovine serum albumin in Tris-buffered saline with 0.05 % Tween 20 (TBST) for 120 min at room temperature, the plates were washed six times with TBST. One hundred μl of primary antibody, rabbit anti-SoyFERH1 serum (1:4,000), was added and the plates gently shaken for 1 h. Plates were again washed as described above and 100 μl TBST with diluted (1:2,000) goat anti-rabbit IgG conjugated with horseradish peroxidase (Bio-Rad, Hercules, CA, USA) was added as a secondary antibody and the plates were incubated for 60 min. The plates were rewashed as described above, and 200 μl well^−1^ of 3,3′,5,5′-tetramethylbenzidine (TMB) was added. After 30 min incubation at room temperature, the reaction was stopped by adding 100 μl of 3 M HCl. The optical density (OD) of each reaction was measured at 450 nm.

For in situ Western hybridization, mature polished seeds were soaked in distilled water at 4 °C overnight. The softened seeds were sectioned longitudinally with a razor blade. The Qu et al. (2005) protocol was followed for SoyFERH1 distribution in rice seeds.

Western blot analysis was carried out as described previously (Datta et al., 1997) using rabbit anti-SoyFERH1 serum.

### Iron staining for localization of Fe

Perls’ Prussian blue technique was employed for the localization of Fe. Polished whole rice seeds were soaked in water in a 1.5-ml microfuge tube overnight. Seeds were sectioned in a Petri dish using a ceramic knife. Whole and sectioned seeds were stained with 2 % HCl (Merck, Germany) and 2 % potassium ferrocyanide (Sigma, MO, USA) to form an insoluble blue color after reaction with Fe. The seeds were washed six times with water to remove excess stain. Observation and documentation were done with an Olympus SZX-7 stereomicroscope with digital camera DP 71 under bright-field mode.

### Gene expression using real-time PCR

Gene expression analysis of the ferritin gene (*SoyFERH1*) was conducted at the milky and mature seed stages of transgenic indica mega-variety IR64. RNA extraction from full grain, primer design, and conditions were optimized for quantitative PCR (qPCR). The relative expression of ferritin genes was normalized using the housekeeping genes *actin*, *ATPase*, and *GAPDH*. Measurements were made in four biological and four technical replicates.

Gene expression analysis was employed to study the changes in expression due to the insertion of the soybean ferritin gene on the endogenous ferritin gene along with Fe transporter genes in seeds of transgenic *indica* cv IR64. Quantitative PCR was performed using primers designed for *FRO1*, *FRO2*, *YSL18*, *SOYFERH1*, *NAAT*, *OsFer*, *YSL15*, *NAS1*, *NAS2*, and *NAS3* (Supplementary Table 1). A real-time PCR was prepared using a master mix of the following reaction components: 6 μl molecular biology grade PCR water, 1 μl forward primer (0.25 μM), 1 μl reverse primer (0.25 μM), 10.0 μl LightCycler^®^ 480 SYBR Green I Master (Roche Diagnostics, Roche Applied Science), and 2 μl cDNA (50 ng reverse-transcribed DNA from total RNA, Roche Diagnostics) were added as a PCR template.

**Table 1:** Transformation efficiencies and molecular analysis data of single transformed (A) and co-transformed (B) IR64 with different promoter::ferritin gene constructs

Construct		T0		Single copy (%)
Gene	Promoter	Total	Transformation efficiency (%)^a^	
(A)				
*SoyFERH1*	*GLUB1*	148	20.8	71.3
*SoyFERH1*	*GLUB4*	63	10.8	75.0
*SoyFERH2*	*GLUB1*	179	38.1	50.5
*OsFER1C*	*GLUB1*	55	18.3	75.0
*OsFER1C*	*GLUB4*	132	14.3	75.5
*OsFER2C*	*GLUB4*	57	19.5	56.8
*OsFER2C*	*GLUB1*	57	16.0	86.7

Construct		T0		Single copy (%)	T1	
Gene	Promoter	Total	Transformation efficiency (%)^a^		Total	G+/H−^b^ (%)
(B)						
*SoyFERH2*	*GLUB1*	79	11.2	43.5	774	24.5
*SoyFERH1*	*GLUB1*	149	3.3	85.7	889	11.7
*SoyFERH1*	*GLUB4*	44	25.9	31.3	459	7.2

^a^Transformation efficiency was computed based on percentage of PCR-GOI-positive plants over number of transferred immature embryos

^b^Marker-free genotype

## Results

### Promoter selection

Three different endosperm-specific promoters, *GLUB1*, *GLUB4,* and *GLB1,* were evaluated for their specific expression in mega-variety IR64. The promoters were fused to the *GUSA* reporter gene to study their expression pattern. The aim was to obtain promoters that are primarily active during early embryo development to allow translocation of Fe.

GUS expression in the seed was observed only in the endosperm in all three promoters evaluated (Supplementary Fig. 1). Based on blue staining of several seeds, it was observed that, at the mature stage, expression was comparable in all three promoter constructs, but, for *GLUB1*-*GUS* seeds, a higher intensity of blue color was observed in the outer part of the endosperm, while *GLB1* had a more even distribution of blue color in the endosperm, similar to the observation of Qu and Takaiwa (2004). At the early developmental dough stage, *GLUB1* showed the highest expression of the *GUS* gene, followed by *GLUB4* and *GLB1*. We selected the first two promoters for further study, based on the hypothesis that highly active promoter during early stage of grain formation will increase the chances of metal loading in the grain.

### Production of transgenic plants with ferritin genes

A large number of independent transgenic events were produced from the integration of different combinations of two rice glutelin promoters with soybean and rice ferritin genes into IR64 with transformation frequency ranging from 10.8 to 38.1 % for single transformation and from 3.3 to 25.9 % for co-transformation (Table 1a, b). A total of 693 and 361 transgenic events from single and co-transformation experiments, respectively, were produced, and integration of the different ferritins was confirmed by PCR analysis with the specific corresponding primers. The percentage of single-copy insertions, analyzed by DNA blotting, was 31–87 % of the PCR-positive plants (Supplementary Fig. 2). Plants were grown to maturity and progenies of independent fertile single-copy transgenic lines of IR64 developed from different constructs were selected and grown in the screenhouse for mass screening to evaluate their Fe concentration.

PCR analysis in the T1 generation of selected co-transformed plants for the presence of the ferritin and absence of *HPT* genes showed that the marker-free genotype comprised about 7–25 % of the segregating progenies among the lines analyzed (Table 1b). Plants having both genes comprised about 50–90 % of the segregating progenies.

### Phenotypic evaluation of transgenic plants

A total of 1,860 plants from 58 T1 IR64 single transformation transgenic events and 768 progenies from 27 co-transformation events with *GLUB1*:*SoyFERH1*, *GLUB4*:*SoyFERH2*, *GLUB1*:*SoyFERH2*, *GLUB1*:*OsFER1C, GLUB1*:*OsFER2C,* or *GLUB4*:*OsFER2C* were transplanted in the paddy soil screenhouse (Supplementary Fig. 3), consisting of seven T1-positive plants/events and three corresponding nulls (azygous) in three replicates. Agronomic characteristics, including fertility, plant height, seed weight, panicle number, and panicle length of the plants, were measured and evaluated at three different growth stages. Variation between mean measurements of the five parameters was analyzed statistically using SAS. The mean values of seed weight per plant and plant height among transgenics (single transformation) with different ferritin genes, their null segregants, and the wild-type IR64 were not significantly different at the 5 % level of significance (Table 2). The mean values of tiller number, panicle length, and panicle number increased in transgenics, and in their nulls. For co-transformation transgenic plants, the mean values of seed weight, plant height, and panicle length were not significantly different while the mean values of the tiller number and panicle number increased in the transgenics and their nulls (Table 2).

**Table 2:** Statistical analysis of agronomic characteristics of transgenic events in IR64 background using different constructs encoding for the ferritin gene and its null counterpart

Plant type	Seed weight per plant		Plant height		Tiller number		Panicle length		Panicle number	
	*n*	g	*n*	cm	*n*	no.	*n*	cm	*n*	no.
*Single transformation*										
IR64 wild type	474	13.15 ± 0.29A	354	106.71 ± 2.72A	354	8.05 ± 0.18B	1,657	23.21 ± 0.08B	354	8.15 ± 0.22B
*GLUB1*::*SoyFERH1*										
T	131	14.94 ± 0.53A	97	101.56 ± 0.48A	97	10.99 ± 0.36A	484	25.02 ± 0.48A	97	10.81 ± 0.35AB
N	48	15.99 ± 0.93A	38	101.33 ± 0.81A	38	9.87 ± 0.49AB	189	24.46 ± 0.23AB	38	9.69 ± 0.50AB
*GLUB4*::*SoyFERH1*										
T	165	14.38 ± 0.43A	144	100.17 ± 0.59A	144	9.99 ± 0.26AB	715	23.50 ± 0.12AB	144	9.79 ± 0.26AB
N	69	14.72 ± 0.76A	60	99.13 ± 1.21A	60	9.71 ± 0.45AB	298	23.78 ± 0.18AB	60	9.66 ± 0.45AB
*GLUB1*::*SoyFERH2*										
T	140	12.60 ± 0.62A	111	99.12 ± 0.66A	111	10.57 ± 0.39AB	552	24.43 ± 0.48AB	111	10.43 ± 0.39AB
N	54	13.30 ± 0.95A	44	99.76 ± 0.80A	44	9.61 ± 0.50AB	218	24.01 ± 0.25AB	44	9.40 ± 0.49AB
*GLUB1*::*OsFER1C*										
T	160	15.60 ± 0.62A	146	100.54 ± 0.69A	146	10.92 ± 0.32A	718	23.93 ± 0.19AB	146	10.81 ± 0.32AB
N	63	15.93 ± 1.05A	57	101.32 ± 0.79A	57	9.93 ± 0.45AB	282	23.89 ± 0.21AB	57	9.79 ± 0.46AB
*GLUB4*::*OsFER1C*										
T	144	14.61 ± 0.54A	133	98.65 ± 0.49A	133	11.80 ± 0.32A	664	23.87 ± 0.33AB	133	11.66 ± 0.34A
N	60	16.30 ± 0.92A	54	97.96 ± 1.09A	54	11.67 ± 0.81A	265	23.88 ± 0.23AB	54	12.51 ± 1.92A
*GLUB1*::*OsFER2C*										
T	104	17.00 ± 0.71A	90	102.01 ± 0.40A	90	11.54 ± 0.40A	449	24.40 ± 0.13AB	90	12.00 ± 0.75A
N	44	17.63 ± 1.19A	38	102.08 ± 0.61A	38	11.27 ± 0.63AB	188	24.24 ± 0.26AB	38	11.02 ± 0.63AB
*GLUB4*:: *OsFER2C*										
T	164	16.01 ± 0.52A	147	102.49 ± 0.30A	147	10.46 ± 0.28AB	727	24.51 ± 0.12AB	147	10.24 ± 0.28AB
N	67	17.30 ± 1.00A	60	102.09 ± 0.43A	60	10.60 ± 0.48AB	294	24.64 ± 0.17AB	60	10.39 ± 0.47AB
*Co*-*transformation*										
IR64 wild type	75	13.43 ± 1.98A	28	99.47 ± 0.97A	103	10.76 ± 1.94B	141	21.44 ± 1.99A	98	10.67 ± 1.83B
*GLUB1*::*SoyFERH2*										
T	42	12.57 ± 1.92A	29	99.04 ± 0.93A	57	16.96 ± 1.54A	112	22.12 ± 1.9A	63	16.36 ± 1.97A
N	8	11.55 ± 1.71A	4	99.90 ± 0.55A	9	14.11 ± 1.83AB	20	21.02 ± 1.76A	9	14.01 ± 1.80AB
*GLUB4*::*SoyFERH1*										
T	9	17.96 ± 1.52A	3	98.00 ± 1.73A	3	17.02 ± 2.00A	4	25.50 ± 1.20A	4	14.75 ± 4.27AB
N	6	13.72 ± 1.60A	0	NO DATA	0	NO DATA	0	NO DATA	0	NO DATA
*GLUB1*::*SoyFERH1*										
T	16	13.51 ± 1.96A	5	99.80 ± 1.30A	12	15.67 ± 1.92AB	26	21.70 ± 1.96A	12	15.67 ± 1.92A
N	34	13.24 ± 1.95A	24	100.41 ± 1.93A	34	13.16 ± 1.89AB	68	21.62 ± 1.99A	35	13.37 ± 1.97AB

For each column, values of treatment means followed by a common letter are not significantly different at 5 % level of significance. *n* = sample size, *T* = transgenic, *N* = null

### Grain quality evaluation of high-iron seeds

The grain quality of three events each from single transformation and co-transformation transgenic plants representing four different phytoferritin genes was found to be similar in milling and cooking quality to that of mega-variety IR64 in our grain quality evaluation study (Supplementary Table 2). The amylose content of the transgenic events analyzed ranged from low to intermediate. The gelatinization temperature of the transgenic seeds was scored as intermediate to high-intermediate. The transgenic and wild-type seeds had soft gel consistency, ranging from 85 to 100 mm in length. The protein content of the transgenic seeds was 10.1–12.6 %. In terms of milling characteristics, the milling potential score of the transgenic seeds was 1 and chalkiness ranged from 1 to 2, which is similar to that of the IR64 wild type.

### Effect of the promoter and transgene on Fe concentration in the endosperm

Seeds from individual plants of 58 events of the T1 generation were polished by a Kett mill and analyzed for Fe concentration using a low-cost colorimetric assay, followed by ICP-OES at the IRRI Analytical Service Laboratory (ASL) for representative samples. Milled rice from 16 individual plants from each independent event was pre-screened for Fe content using a colorimetric assay. The Fe concentration of milled rice of selected events from each construct was then analyzed by ICP-OES. Supplementary Table 3 shows the comparison of the Fe concentration of IR64 plants expressing ferritin genes in the T2 generation driven by *GLUB1* (5.63 ± 0.67 mg kg^−1^) and plants with ferritin genes regulated by *GLUB4* (6.21 ± 0.74 mg kg^−1^). Fe concentration by colorimetric assay for transgenic plants with the *OSFER2C* gene was of a level similar to that of the wild-type IR64 (data not shown); these lines were therefore not advanced further for further analysis and evaluation.

Forty-five events were selected for the T2 homozygous screening. Homozygous lines were selected based on segregation analysis. We changed the polishing method to the Genogrinder (Bautista et al. 2004) in the succeeding generations to be more stringent in milling quality for a low number of seeds. The Fe concentration of wild-type IR64 using this instrument was consistently 2–3 mg kg^−1^, which is similar to observations by Batista et al. (2012) that Fe concentration of IR64 in several commercial mills ranged from 2 to 3 mg kg^−1^. Both *GLUB1* and *GLUB4* promoters showed a comparable increase in Fe in the transgenic seeds (Supplementary Table 3). In addition, ICP results of the T2 and T3 generation of transgenic seeds differed between different types of ferritin genes (*OsFER1C*, *SoyFERH1,* and *SoyFERH2*). However, the Fe concentration of transgenic seeds from *OsFER1C*- and *SoyFERH2*-transformed plants was significantly lower in the T3 ICP results than in plants transformed with *SoyFERH1* (Supplementary Fig. 4).

**Fig. 1:**
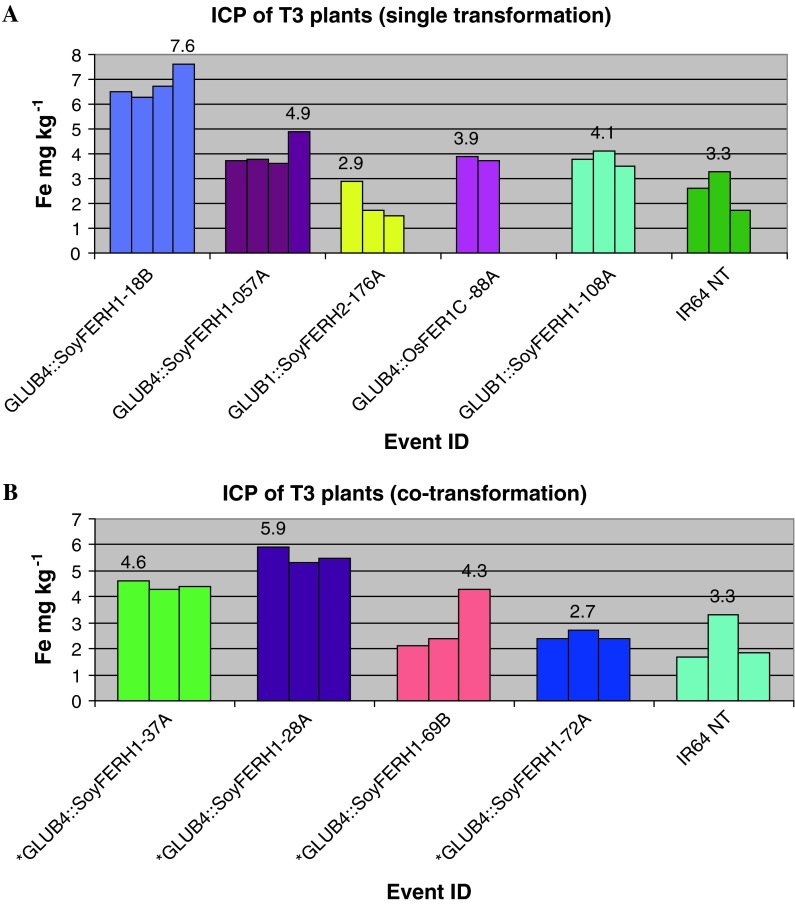
Iron concentration in single transformation (**a**) and co-transformation (**b**) transgenic plants in T3 generation. *Co-transformation transgenic plants. Each *bar* represents a biological replicate of the event

Fe concentration in the polished transgenic seeds of the T3 generation of single and co-transformation events representing different constructs was measured using ICP at the Waite Analytical Laboratory in Adelaide (Fig. 1). The maximum value of Fe concentration in transgenic lines expressing the ferritin gene in homozygous lines was 7.6 mg kg^−1^ for *GLUB4*::*SoyFERH1* (single transformation), followed by 5.9 mg kg^−1^ for *GLUB4*::*SoyFERH1* (co-transformation). The transgenic T3 lines showed as much as a 3.4-fold increase in Fe concentration compared with the non-transgenic seeds.

### Correlation of Fe concentration and ferritin expression

**Fig. 2:**
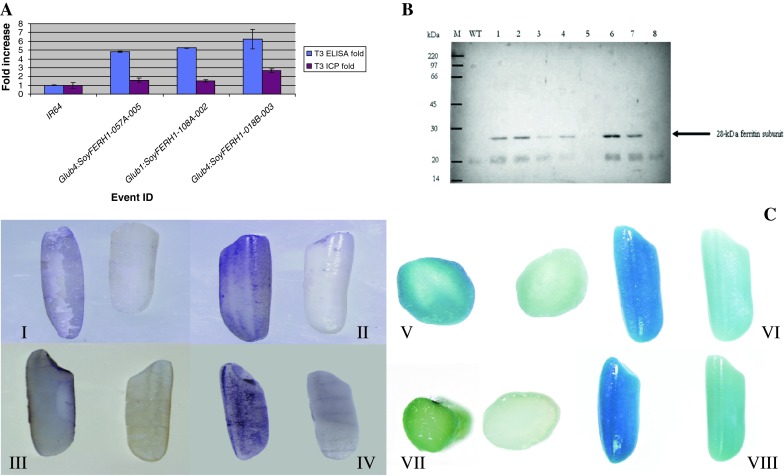
Expression of ferritin genes. **a** Comparison of fold increase in *SoyFERH1* protein and Fe concentration in transgenic plants in T3 generation ELISA (*blue bars* represent mean fold change ± standard error obtained from three technical replicates). Data are illustrated as fold change in transgenic seeds relative to baseline values (wild-type IR64) Fold increases in Fe content (*magenta bars* represent mean fold change ± standard error) in seeds of transgenic plants over that of wild type were measured from three biological replicates. **b** Western blot analysis of *SoyFERH1* ferritin in transgenic rice seeds. Fifty micrograms of total proteins extracted from each transgenic and non-transgenic seed were fractioned by SDS-PAGE, immunoblotted, and then bound with soybean ferritin rabbit polyclonal antibodies. The predicted 28-kDa protein band is ferritin. *M* = Protein Size Marker (Amersham, RPN 756); *WT* = wild-type IR64; *1*–*4*, *6*–*7* = T1 IR64 events with *SoyFERH1*; *5* and *8* are null segregants. **c** In situ Western blot of transgenic rice seeds with ferritin. (I–II) Polished seed (longitudinal section and whole seed) of IR64 transformed with *SoyFERH1* driven by *GLUB4* promoter bound with anti-*SoyFERH1* antibody (*left*) versus polished IR64 seed (*right*). (III–IV) Polished seed (longitudinal section and whole seed) of marker-free IR64 transformed with *SoyFERH1* driven by *GLUB1* promoter bound with anti-*SoyFERH1*antibody (*left*) versus polished IR64 seed (*right*). (V–VI) Fe localization using Pearl Prussian blue staining (transverse section and whole polished seed of transgenic IR64 with *SoyFERH1* driven by *GLUB4* (*left*) versus wild-type IR64 (*right*). (VII–VIII) Fe localization using Pearl Prussian blue (transverse section and whole polished seed of transgenic marker-free IR64 with *SoyFERH1* driven by *GLUB1* (*left*) versus wild-type IR64 (*right*)

SoyFERH1 protein expression in T2 and T3 seeds of a number of selected events driven by *GLUB1* and *GLUB4* promoters was studied by ELISA, using rabbit anti-SoyFERH1 antibody. The ELISA fold values were calculated using IR64 wild type as background value and were compared with the Fe ICP values. Transgenic events with higher ferritin expression in ELISA showed higher Fe using ICP in both the T2 (data not shown) and T3 generations (Fig. 2a).

### Ferritin localization and iron distribution in transformed lines

The expression pattern of soybean ferritin directed by a rice endosperm-specific promoter (*GLUB1* or *GLUB4*) was determined by in situ Western hybridization. In IR64 plants with *SoyFERH1* driven by *GLUB4*, color development was more homogeneous in the endosperm with higher concentrations in its outer cells (Fig. 2c I–IV). In IR64 transgenic T2 and T3 plants with *SoyFERH1* directed by the *GLUB1* promoter, ferritin was expressed in a localized part of the starchy endosperm tissue. Non-transformed (NT) rice seed controls remained minimally stained compared with the transgenic lines.

Prussian blue staining clearly shows the accumulation of Fe in the endosperm cells of polished transgenic rice grains (T2 and T3), as indicated by the blue color (Fig. 2c V–VIII). In non-transgenic rice grains, the endosperm showed minimal color development.

Western blot analysis of total protein extracts from mature polished seed from T1 plants, null segregants, and the wild type was performed to investigate the expression of ferritin (Fig. 2b). Polyclonal antibody directed against SoyFERH1 ferritin was bound to a 28-kDa band from transgenic plants transformed with the *SoyFERH1* gene, while no 28-kDa band was observed in proteins from the NT and the null segregants from the T1 trangenic lines. Though ferritin amounts varied between different individual lines, the protein could be observed in almost all the transformants assayed. In addition, another band, about 22 kDa, was observed in transgenic rice, although at a lower intensity in the wild-type and null segregants of the transgenic lines, which could be the native rice ferritin (23–24 kDa).

### Gene expression study of ferritin genes

**Fig. 3:**
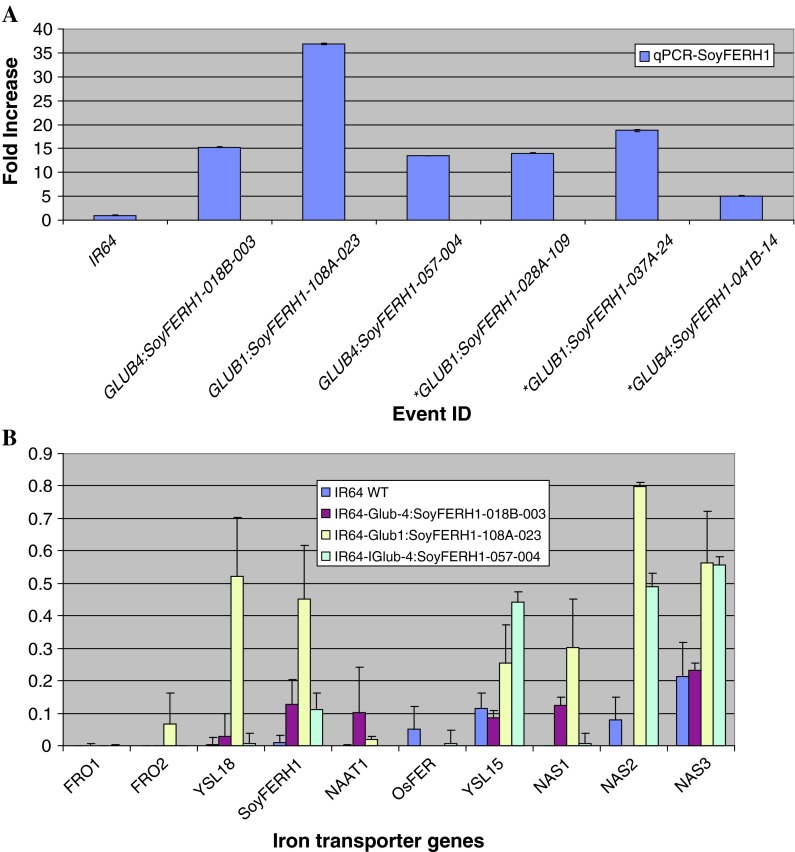
Quantitative PCR measurement of expression of the ferritin gene in transgenic and wild-type IR64 seeds (**a**) and selected Fe homeostasis genes in different transgenic lines compared with wild-type IR64 (**b**) *co-transformation transgenic plants

To examine the expression of the ferritin gene, total RNA isolated from T2 immature seeds of six lines (single and co-transformation events) was analyzed by qPCR using primers specific for soybean ferritin. The increase in expression of the *SoyFERH1* gene of the different events ranged from 3- to 37-fold (Fig. 3a).

The expression of Fe transporter genes *FRO1*, *FRO2*, *YSL16*, *YSL18*, *NAS1*, *NAS2*, and *NAS3* and ferritin genes *SoyFERH1* and *SoyFERH2* was analyzed in selected IR64 transgenic plants and compared with the expression of those genes in IR64 seeds in the mature stage. Expression of the endogenous transporter genes involved in cellular delivery of Fe generally increased in the three transgenic lines, particularly for *NAS2*, *NAS3*, *YSL15*, and *YSL18* genes (Fig. 3b) and downregulation of Fe was observed in the transgenic events.

## Discussion

The high transformation efficiency in both single transformation and co-transformation of mega-variety IR64 allowed the evaluation of multiple lines for agronomic traits and eventual selection of lines with higher iron concentration. Phenotypic data of plants from independent transgenic events with a single insertion of the ferritin gene in paddy soil conditions showed similar seed weight per plant, plant height (for single and co-transformation), and panicle length (co-transformation) among transgenic, null, and wild-type plants, indicating no effect of the genes on these traits. However, variations were observed in tiller number, panicle number, and panicle length (for single transformants) in both transgenic and null segregrants, most likely due to an effect of environment or tissue culture rather than a gene effect. We also did not see any chlorotic symptom in the leaves as reported by Qu et al. (2005).

Grain quality evaluation of seeds from transgenic plants shows that seeds with high Fe are comparable in milling characteristics and cooking traits with non-genetically modified IR64 seeds. IR64 possesses good market value because of its preferred milling properties and good cooking quality; transgenic rice with ferritin genes could thus offer additional nutritional value to this preferred variety. Our results for milling potential and chalkiness with advanced transgenic lines with *SoyFERH1*, *SoyFERH2*, and *OsFER1C* indicate that the transgenic seeds have high milling quality from a marketing standpoint. Based on the adoption rate of IR64 with submergence tolerance in Southeast Asia (Manzanilla et al. 2011), biofortification by the introduction of a transgene into mega-variety IR64 will increase the chances of quicker adoption.

*GLUB1* and *GLUB4* promoters were selected for expressing plant ferritins in transformation to allow translocation of Fe from leaves to the endosperm in an early seed development stage. However, we found that, even though ferritin gene expression could be increased up to 37-fold using *GLUB1* and *GLUB4*, similar to the *GLB1* promoter study of Qu et al. (2005), the maximum Fe accumulation was only threefold that of the wild type. Increasing sink at early grain development stage could therefore only moderately increase mature grain Fe. Gene expression analysis showed that the introduction of the *SoyFER* gene in rice increases gene expression of metal transporters yellow stripe-like (*YSL15* and *YSL18*) and nicotinamine synthase (*NAS2* and *NAS3*). Nicotianamine (NA) is thought to be an essential chelator for metal homeostasis and plays key roles in Fe metabolism and homeostasis in all higher plants. Nicotianamine synthase catalyzes the trimerization of *S*-adenosylmethionine to form one molecule of NA. Apparently, an increase in Fe storage protein gives a signal to other Fe homeostasis genes to increase the rate of Fe transport to the seed; in other words increasing the sink upregulates the genes involves in mobilizing the source, but a decrease in native ferritin levels was observed. However, despite the high availability of storage protein in the grain and increased expression of the Fe homeostasis gene in the grain, limited additional Fe was loaded to the grain, showing the need for stronger mobilization of Fe from vegetative tissue to the grain.

Free Fe in cells is toxic and strict control of Fe homeostasis is required to avoid deficiency and toxicity. Ferritin plays a role in both Fe housekeeping and storage and also in Fe detoxification. Moreover, the physiological role of endogenous ferritin appears to be more related to protection against excess Fe than to reserve storage (Ravet et al. 2009a).

We report the generation of marker-free transgenic rice carrying ferritin genes driven by endosperm promoters, and its evaluation for increased Fe in the seed and agronomic analysis. Stable inheritance was confirmed by progeny analysis of the ferritin genes according to the Mendelian (3:1) ratio. PCR analyses of T1 plants show 7–25 % marker-free plants with high Fe (5.9 mg kg^−1^) in the seeds. This indicates that co-transformation using two *Agrobacterium* strain individually harboring the *HPT* or ferritin genes resulted in integration in different loci of the two genes in most co-transformation events. This separation allowed the segregation of the genes in the next generation as planned. Genetically modified plants without antibiotic-selectable markers are likely to be more acceptable by the public, and also with lower numbers of transgenes there would be a reduced requirement for biosafety evaluation of the novel protein in the transgenic plant. In the genetic background of IR64, a popular *indica* variety with Fe concentration 2–3 mg kg^−1^, we obtained a concentration based on ICP as high as 7.2 ± 1.52 mg kg^−1^ in single-copy T2-generation polished seed (data not shown), slightly higher than the Fe concentration previously reported by Wirth et al. (2009), with 7 mg kg^−1^ in events with single-copy insertion. This accounts for a 2.5-fold increase in extra Fe compared to the control. The highest Fe concentration of a T3 transgenic IR64 event expressing the ferritin gene (7.6 mg kg^−1^) was obtained from *GLUB4*::*SoyFERH1* (single transformation-homozygous line). A transgenic line with *SoyFERH2* driven by *GLUB1* had an Fe concentration of 2.9 mg kg^−1^. *SoyFERH1*- and *SoyFERH2*-transformed lines had much higher Fe concentration than the line with the *OsFER1C* gene. In co-transformation lines, the highest Fe concentration obtained was 5.9 mg kg^−1^ from one of the *GLUB4*::*SoyFERH1* plants. Sequence comparison of two soybean and two rice ferritins showed high similarity in the α, β, and D helical regions but they differed in the C helical region (Supplementary Fig. 5). In addition to this difference, the transit peptide region of the sequences showed only low sequence similarity. This may also cause a difference in their ability to accumulate Fe in the grain. Introduction of an extra copy of endogenous rice ferritin can also trigger post-transcriptional gene silencing (Vaucheret et al. 2001) due to the specific degradation of a population of homologous RNAs, and result in a minimal increase in or even reduction of the ferritin content. The extra copy of endogenous ferritin may also cause tighter control of Fe homeostasis compared with exogenous ferritin, since one major role of ferritin is to reduce Fe-mediated oxidative stress, apart from functioning as a Fe storage protein (Ravet et al. 2009a, b).

The efficacy of *GLUB1* and *GLUB4* endosperm-specific promoters, both more active in the early stage of grain filling, in increasing ferritin expression and subsequently Fe concentration in polished grain is not consistently different among different generations of different constructs, based on ICP and spectrophotometric quantification and ELISA using *SoyFERH1* antibody.

The high-Fe lines consistently showed high protein expression using ELISA. Transgenic lines with a six to sevenfold increase in ferritin protein expression determined by ELISA exhibited an average threefold increase in Fe concentration as determined by ICP-OES. Aluru et al. (2011) also reported a strong correlation between ferritin concentration and Fe content in transgenic maize with soybean ferritin. Qu et al. (2005) observed that the fold increase in the amount of ferritin storage estimated using Western blotting was not linear with the increase in Fe concentration. From the population analyzed in this study, the lines expressing higher ferritin had higher Fe.

Iron translocation from leaf tissue and other plant tissues to seed endosperm is known to be one of the major limiting factors in a biofortification approach. Iron is incorporated into the ferritin shell to form the mineral core in the plastids (Waldo et al. 1995). Although the concentration of Fe measured in elite transgenic IR64 (7 mg kg^−1^) was higher than with the two-gene approach (Wirth et al. 2009), it is clear that increasing the sink should be coupled with an improvement in Fe loading to the amyloplast, where the ferritin is stored in the seed. Goto et al. (1999) stated that proper assembly of the protein is required for import of the subunit into the plastid, as well as for Fe storage function.

Another issue regarding over-expressing ferritin for accumulating Fe in the grain is the function of H1 and H2 subunits, according to Deng et al. (2010). Heteropolymeric ferritin may facilitate plant cell absorption of both ferrous and ferric ions from soil more effectively than homopolymeric ferritin. Both Fu et al. (2010) and Deng et al. (2010) reported that the H2 subunit is more resistant to proteolysis than the H1 subunit, but we saw only a slight increase in Fe with events expressing H1 instead of H2. If H1 and H2 act synergistically, expressing both could result in better Fe absorption.

Preliminary results also show that the introduction of the soybean ferritin gene reduces the expression of endogenous genes. Transgenic events expressing *NAS2* (Johnson et al. 2011) and the activation of *NAS2* and *NAS3* genes (Lee et al. 2009, 2012) have been shown to increase Fe content in rice. These studies strongly support the importance of both Fe homeostasis genes in Fe grain filling. In our study, over-expression of exogenous ferritin by itself did not overcome the limiting rate of Fe uptake to the endosperm; however, *NAS2* is a potential gene for pyramiding with other genes to improve Fe concentration in endosperm since it does not cause any detrimental effect on plant performance. Pyramiding genes have been reported by Masuda et al. (2012) and Wirth et al. (2009), where *OsNAS1* was used in both cases. Our results shows that strong upregulation was detected in *OsNAS2*, which was confirmed by previous studies (Lee et al. 2009 and Johnson et al. 2011); for the multigene gene approach *OsNAS2* and *OsNAS3* may thus have more potential than *OsNAS1*.

In summary, our study demonstrated that the introduction and expression of ferritin genes from soybean and rice under the control of endosperm-specific promoters increased the concentration of iron in polished seeds in an important rice variety, IR64, and in its progenies without compromising the agronomic and grain quality of the transgenic plants. Expressing ferritin from soybean was more effective in increasing the iron content in transgenic rice than over-expression of ferritin from rice. Endosperm-specific promoter *GLUB4* resulted in a higher iron concentration in transgenic plants than *GLUB1*, regardless of phytoferritin genes being expressed. Introduction of the ferritin gene is still a potential approach to develop a transgenic product in combination with other gene/s that will allow additional iron loading in the grain to meet the target of fulfilling a significant part of the estimated average requirement of iron in the human diet.

## Electronic supplementary material

Below is the link to the electronic supplementary material.

Supplementary material 1 (DOCX 2928 kb)
