# Supplementary material for: Large-scale production and evaluation of marker-free indica rice IR64 expressing phytoferritin genes
Source: Mol Breed. 2013 Aug 11;33(1):23–37. doi: 10.1007/s11032-013-9931-z (PMC3890568; doi:10.1007/s11032-013-9931-z)
Supplement: Supplementary file 1 — Supplementary material 1 (DOCX 2928 kb) [file 11032_2013_9931_MOESM1_ESM.docx]

**Supplementary Table 1** Primers used for quantitative PCR

| **Gene** | **Forward primer** | **Reverse primer** |
| --- | --- | --- |
| *OsFER* | AGGGATTCGCCAAATTCTTC | CCTTTCTCAGGATGGTCGAA |
| *NAS1* | AGCGCGGTTGAGAAGGCAGAA | GGCATGTTCCTCGTTTACACTGGC |
| *NAS2* | CGCCAAGAGGCTATCCGTCTGA | GATCAATGCCCAGGCGCCTA |
| *NAS3* | CGAGTGTTCGACTGATCACC | CCGGTGATCTTCTCAACCAA |
| *NAAT1* | TGCTGTGGCAGATCATTTGT | TTGGGTAGCCTGGTCTAGGA |
| *SoyFERH1* | GGGATTTGCCAAGTTCTTCA | TACAATGCATCCCCCTTTTC |
| *YSL15* | TGATCATAGCCTACGCGCTCGG | GCGATGAAGCAGCCAATGCC |
| *YSL18* | GACAAGCGAAGAGGCAAGTC | CGATTCCCACATAAGTTCCG |
| *FRO1* | GGCCACCAAGCAGTCCCTCTTT | CGAAAGGCCCGTCCACAAGA |
| *FRO2* | TCCCTGTTGCCGCTCATTGG | CCCGGGGACGTTTGCAACTT |

**Supplementary Table 2** Grain quality assessment of transgenic IR64 lines

| **Sample ID**  **DESIGNATION** | **Cooking traits** | | | | | **Milling potential**  **score** | |
| --- | --- | --- | --- | --- | --- | --- | --- |
|  | **AC** | **GT** | | **GC** | **PC**  **(%)** | **DB** | **C** |
| IR64 control | Low | | HI/I | Soft | 12.2 | 1 | 1 |
| *Single transformation* | | | | | | | |
| IR64-*GlUB4*:: *SoyferH1*-018B-003 | Low | | I | Soft | 11.6 | 1 | 1 |
| IR64- *GlUB4*::*Osfer1C*-088A-006 | Intermediate | | I | Soft | 12.5 | 1 | 2 |
| IR64-*GlUB1*:: *SoyferH2*-176A-002 | Intermediate | | HI/I | Soft | 12.6 | 1 | 2 |
| *Co-transformation* | | | | | | | |
| IR64- *GlUB1*:: *SoyferH1*-028A-109 | Low | | HI/I | Soft | 12.5 | 1 | 2 |
| IR64- *GlUB1*:: *SoyferH1*-072A-094 | Intermediate | | HI/I | Soft | 10.1 | 1 | 2 |
| IR64-*GlUB1*:: *SoyferH1*-37A-129 | Intermediate | | I | Soft | 11.7 | 1 | 2 |

AC = amylose content: waxy (0-2%), very low (3-9%), low (10-19%), intermediate (20-25%), high (>25%); GT = gelatinization temperature: I = intermediate (70-72 °C), HI = high intermediate (73-74 °C), results are from six replicates; GC = gel consistency scored as 1) very flaky rice with hard gel consistency (length of gel, 40 mm or less); 2) flaky rice with medium gel consistency (length of gel, 41 to 60 mm); and 3) soft rice with soft gel consistency (length of gel, more than 61 mm), results are from two replicates; PC = protein content, with range of 8 to 14 considered as normal or marketable; DB = degree of breakage and C = chalkiness score, both classified using the following scale: 1 = mostly whole translucent grains; 2 = few broken/chalky grains, and 3 = >70% of grains are broken; >50% chalky grains

**Supplementary Table 3** Comparison of average iron concentration of IR64 transgenic plants (single transformants) with ferritin genes driven by *GLUB1* versus *GLUB4* promoter. Average iron concentration for each promoter were obtained from 8 transgenic plants

| **Promoter** | **T2 ICP (mg kg^-1^)** | **T3 ICP (mg kg^-1^)** |
| --- | --- | --- |
| *GlUB1* | 5.63 ± 0.67 | 4.60 ± 1.20 |
| *GlUB4* | 6.21 ± 0.74 | 5.03 ± 1.80 |

IR64:*GLUB1:GUS*

IR64:*GLUB4:GUS*

IR64:*GLB:GUS*

IR64:*35S:GUS*

IR64

**Dough (15 DAF)**

**Mature (30 DAF)**


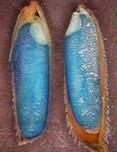

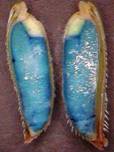

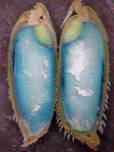

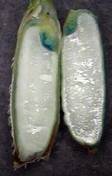

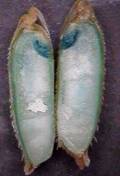

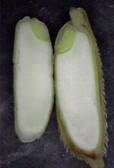

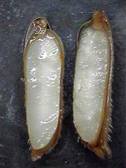

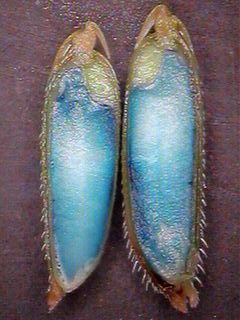

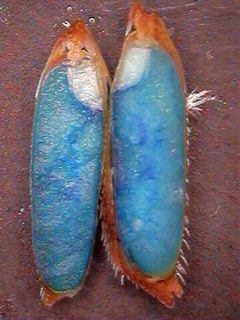

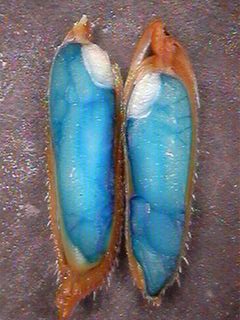


**Supplementary Fig 1** Histochemical *GUS* staining of dough (left) and mature seeds (right) of transgenic IR64 plants with different promoter:*GUS* constructs


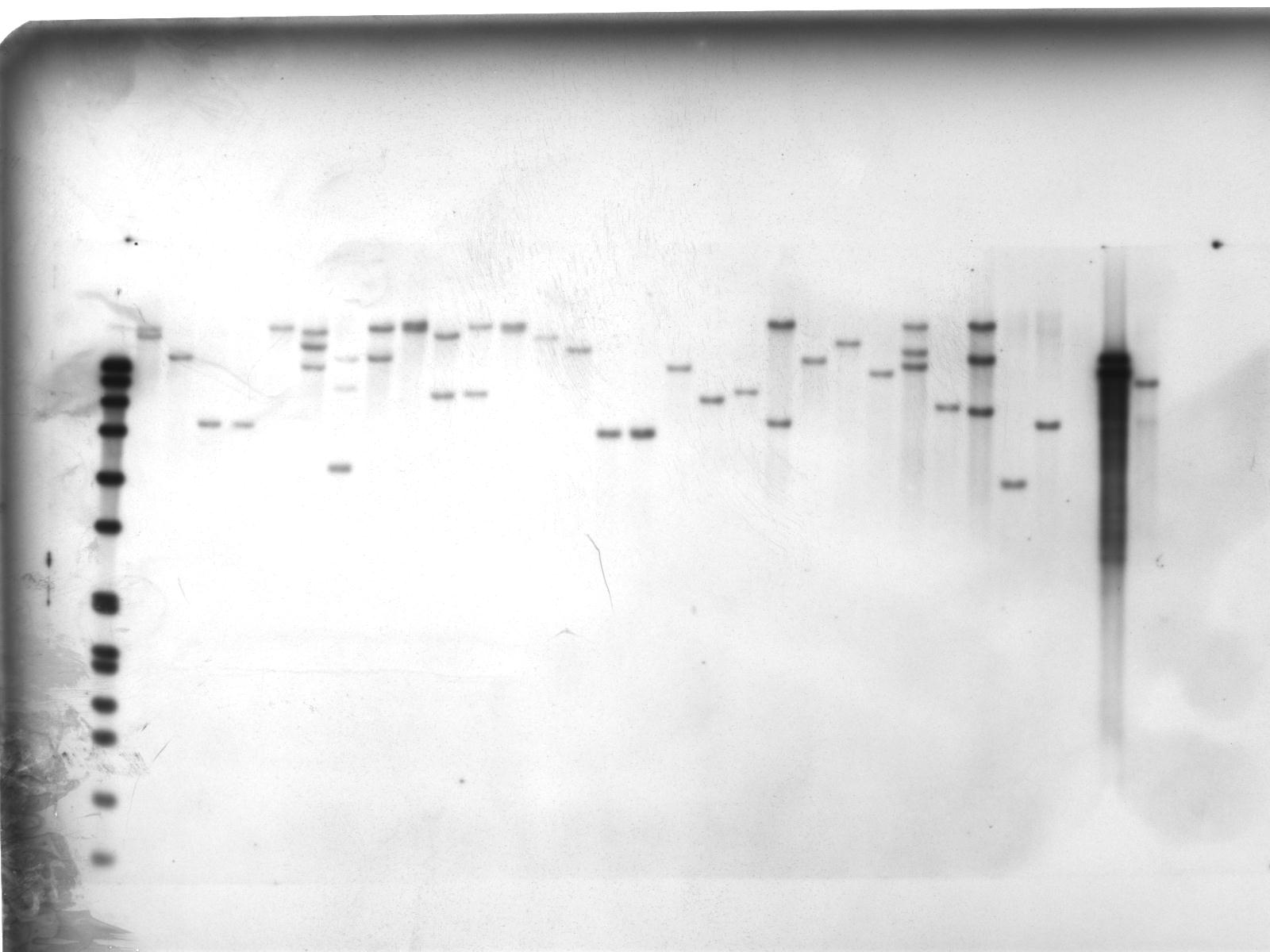


**M 1 2 3 4 5 6 7 8 9 10 11 12 13 14 15 16 17 18 19 20 21 22 23 24 25 26 27 28 WT**

kb

**8.5**

**7.4**

**6.1**

**4.8**

**3.6**

**2.7**

**Supplementary Fig. 2** Southern blot analysis of transgenic IR64 with *SoyFERH1* driven by *GlUB1* promoter. Genomic DNA from each sample digested with *Eco*RI, hybridized with a PCR-labeled probe containing 928-bp fragment of *SoyFERH1* gene. M = Dig-labeled marker VII, WT = wild-type IR64, 1-28 = T0 events with *SoyFERH1*gene

**
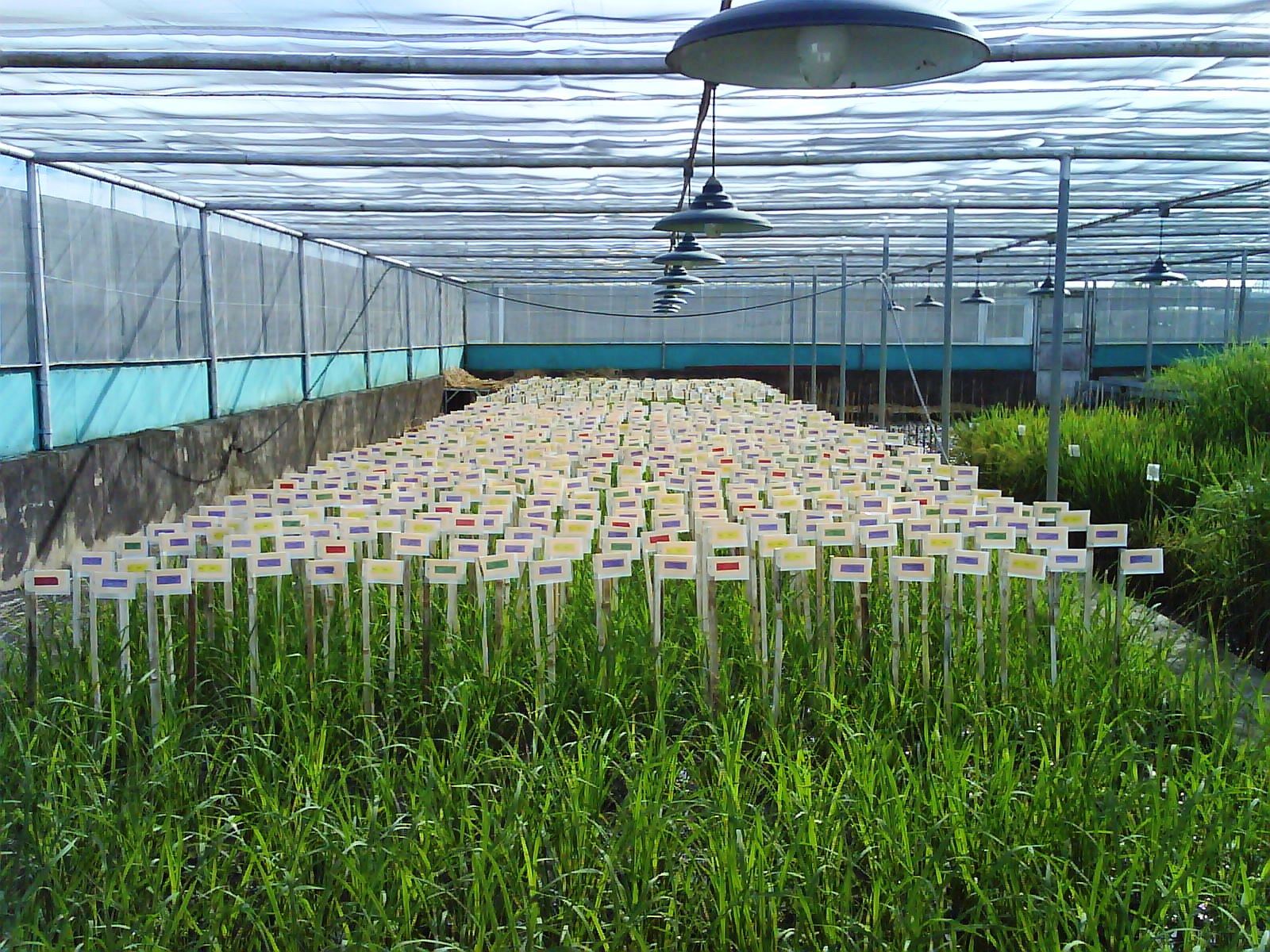
**

**Supplementary Fig. 3** Screenhouse evaluation of phenotype of different T1 transgenic lines

**Supplementary Fig. 4** Comparison of iron concentration of IR64 transgenic plants with different ferritin genes


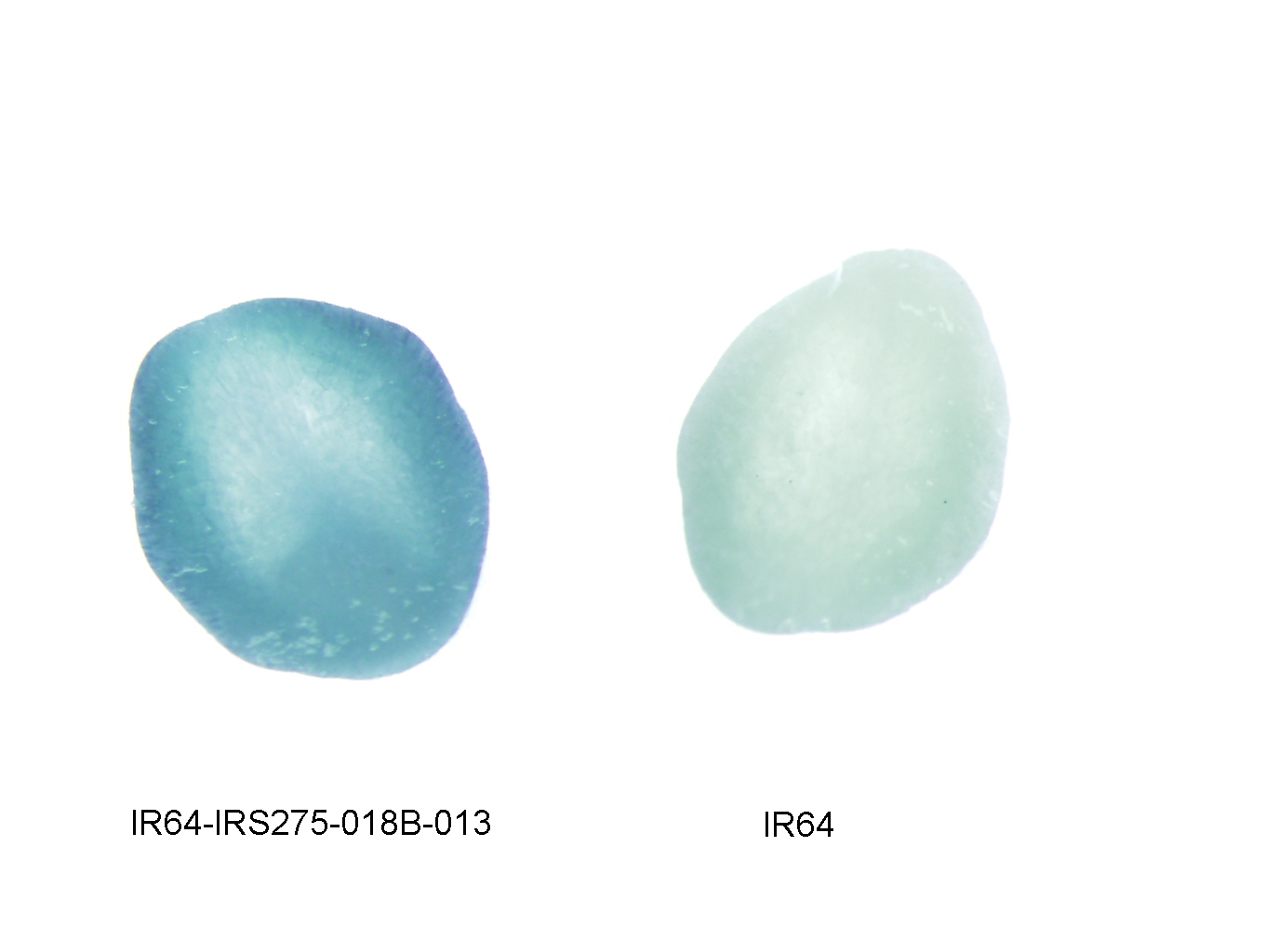


e


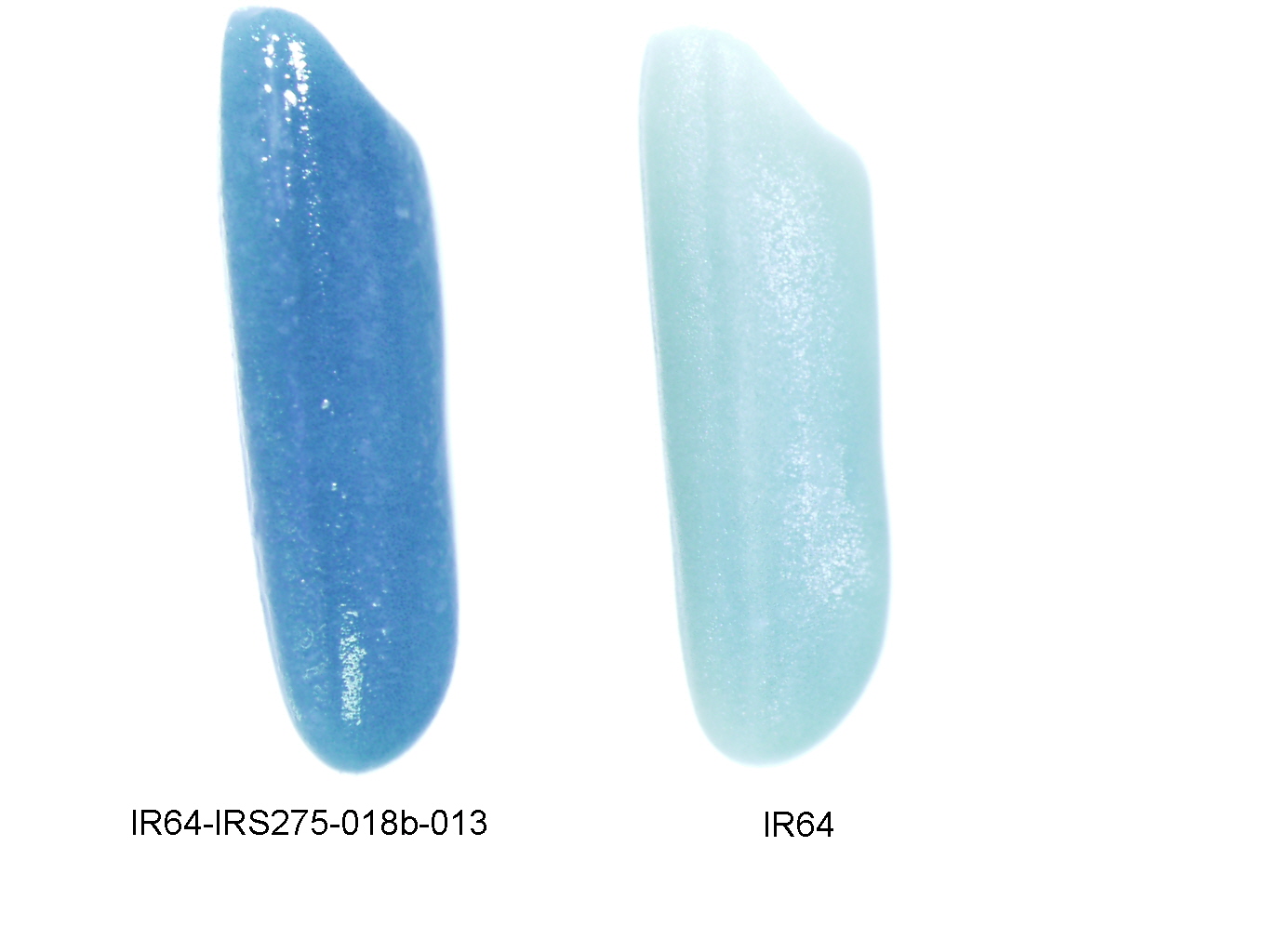


f


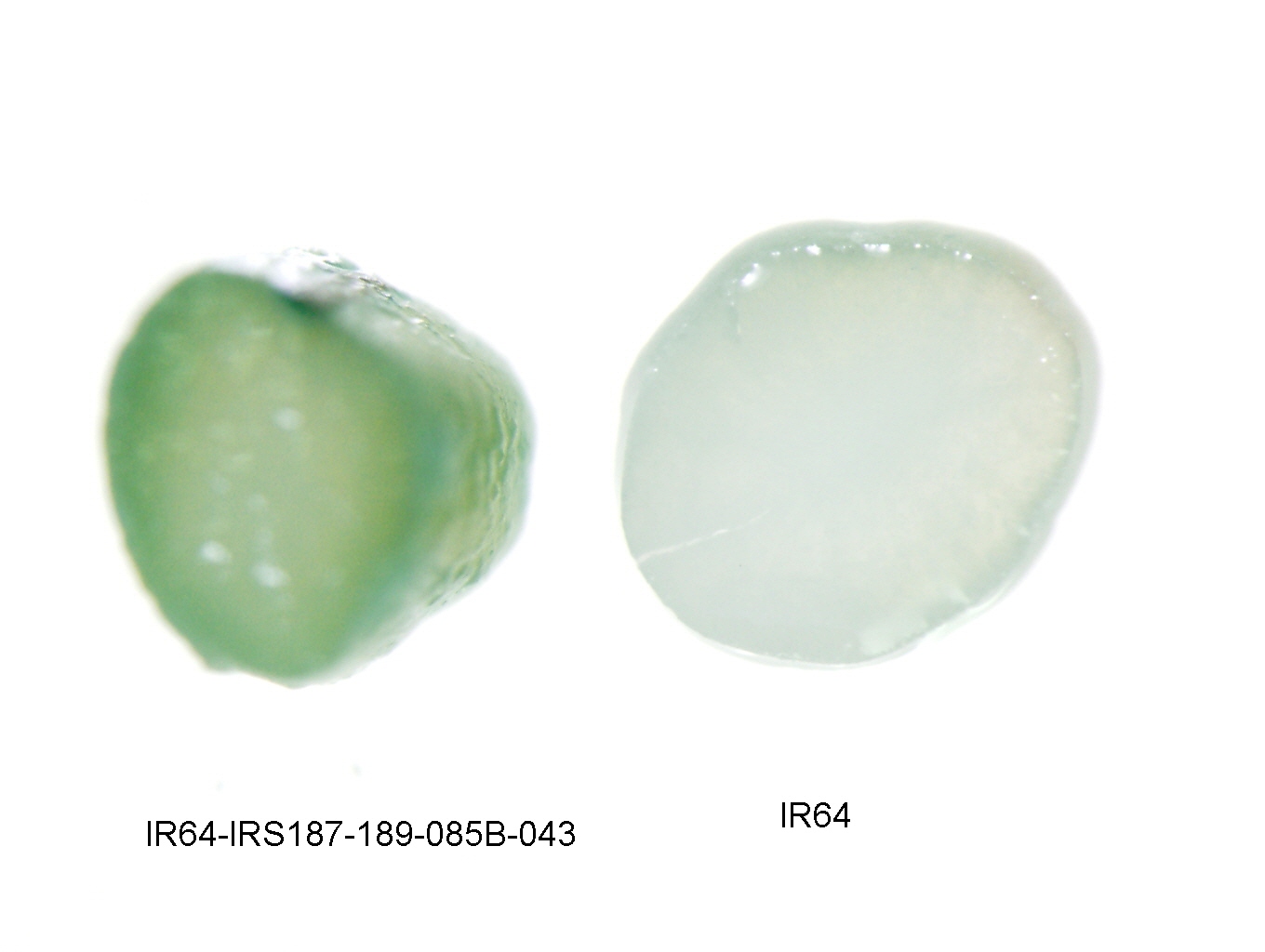

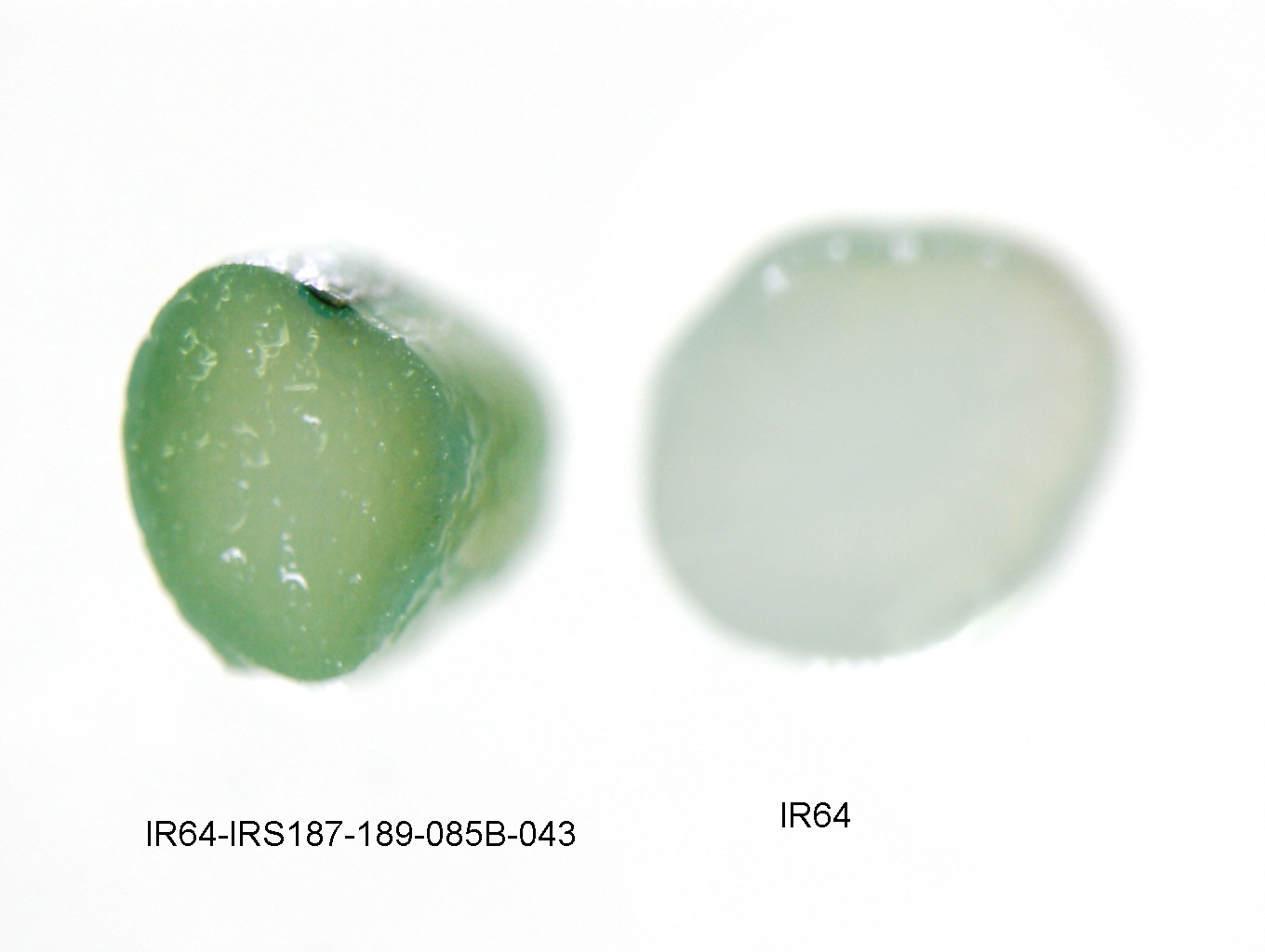


g


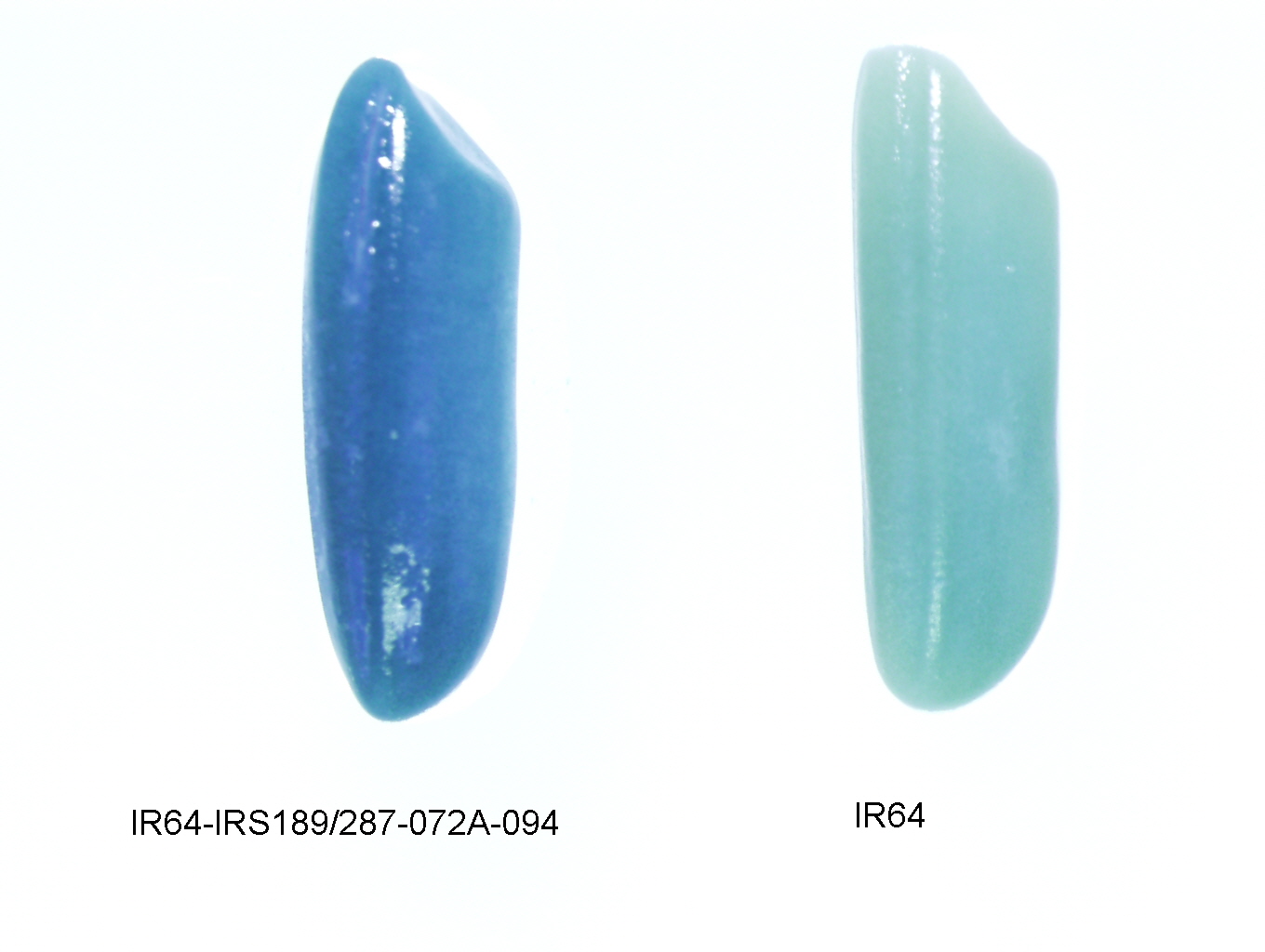


h


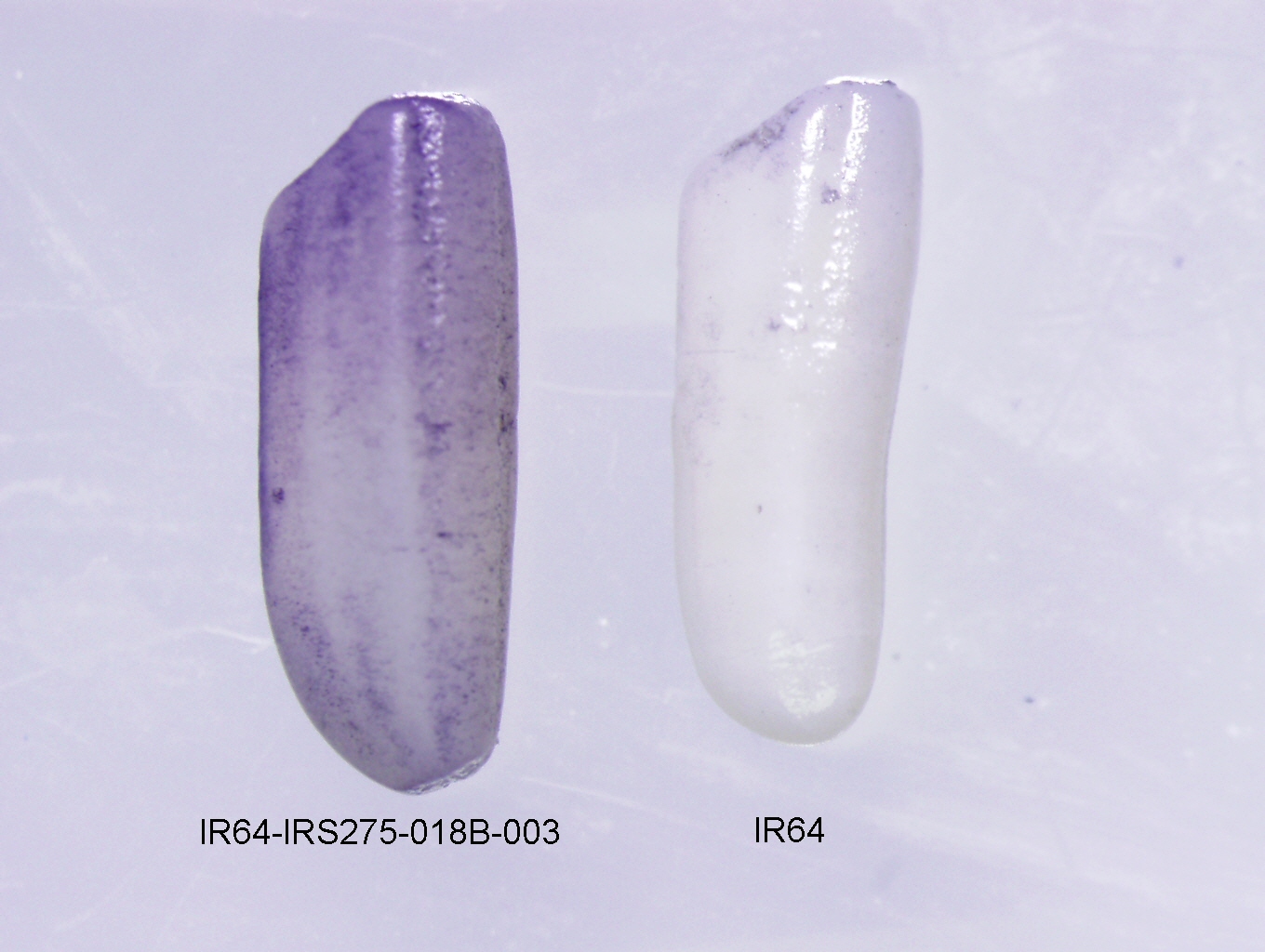

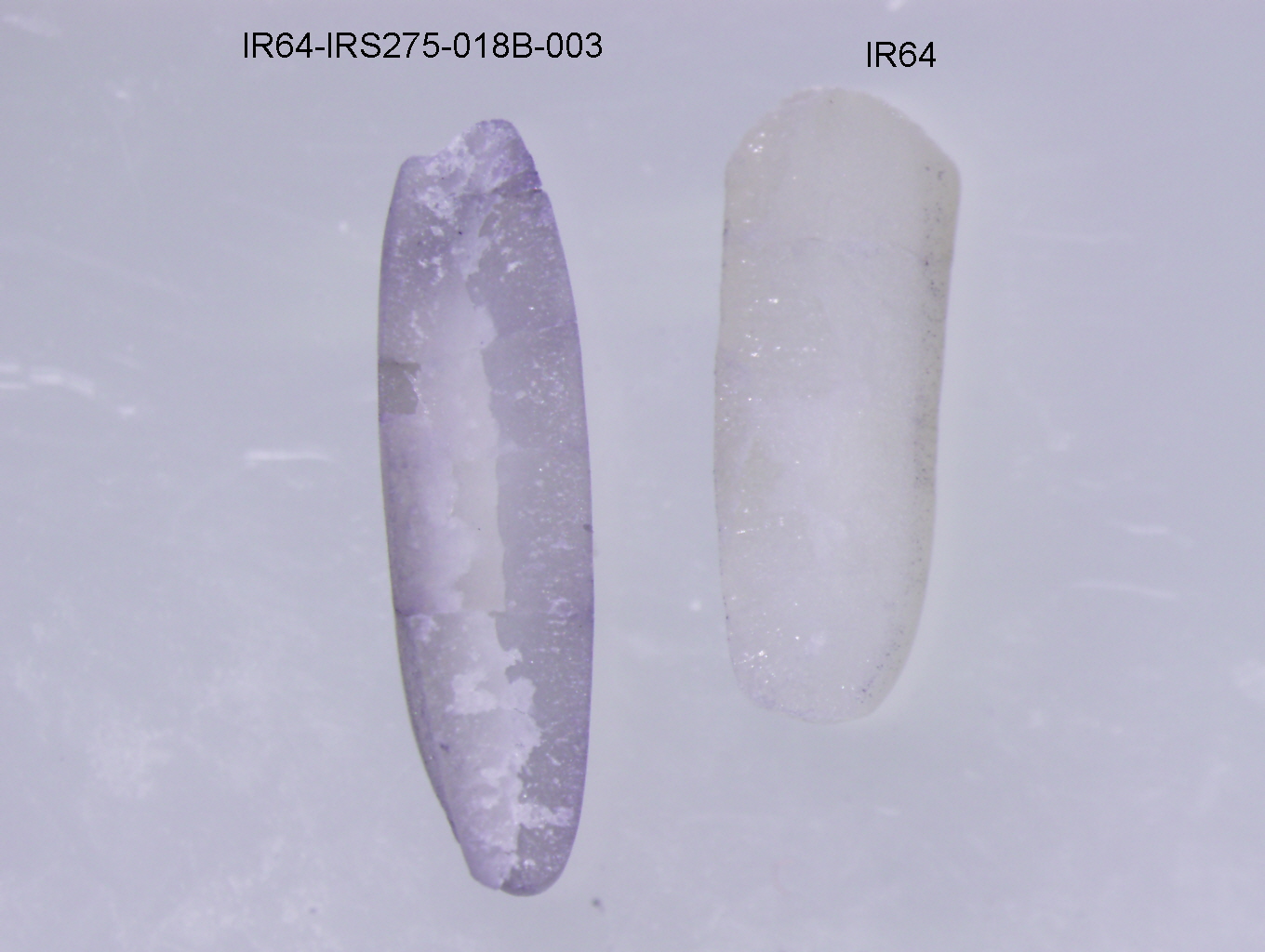

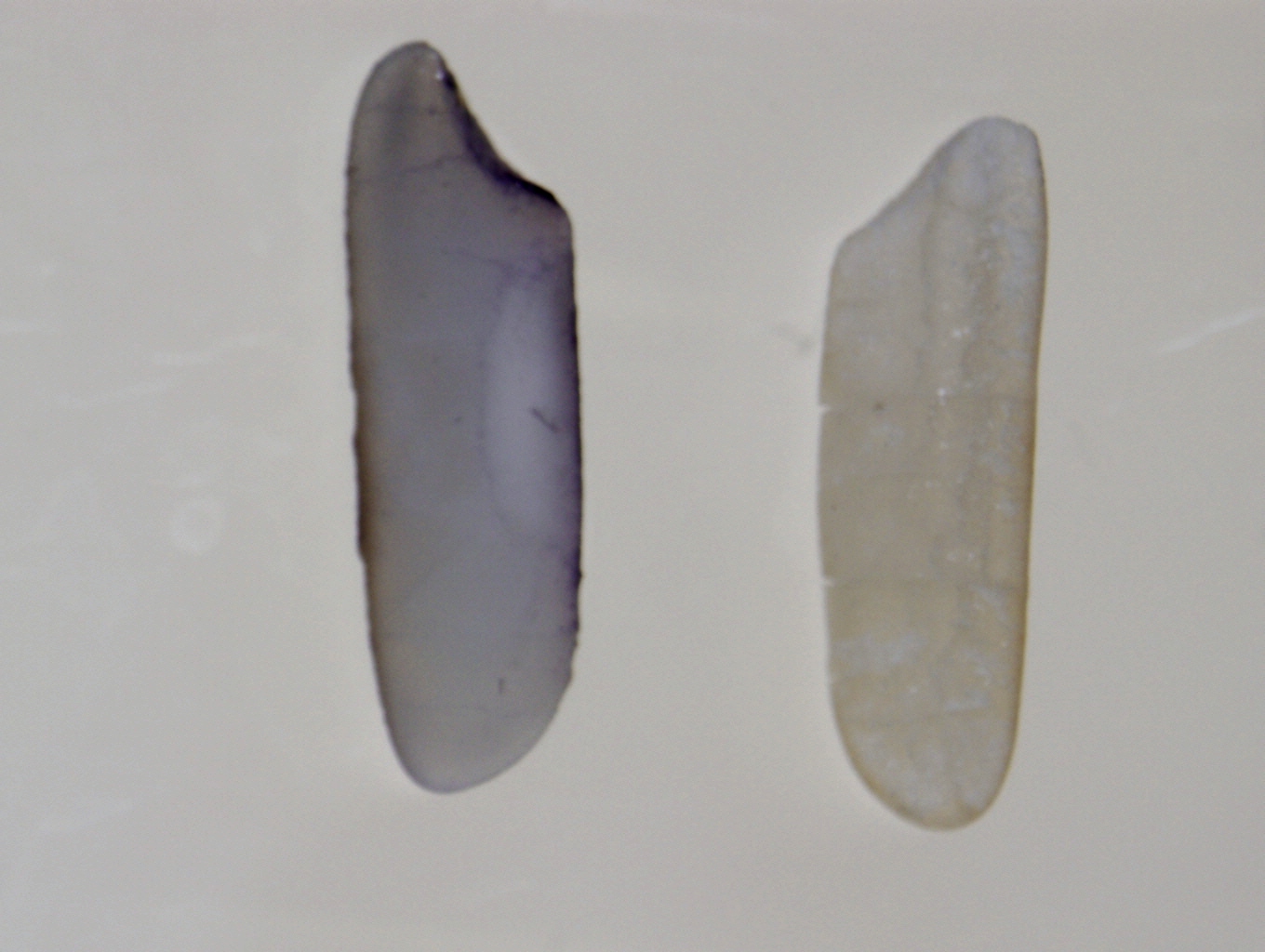

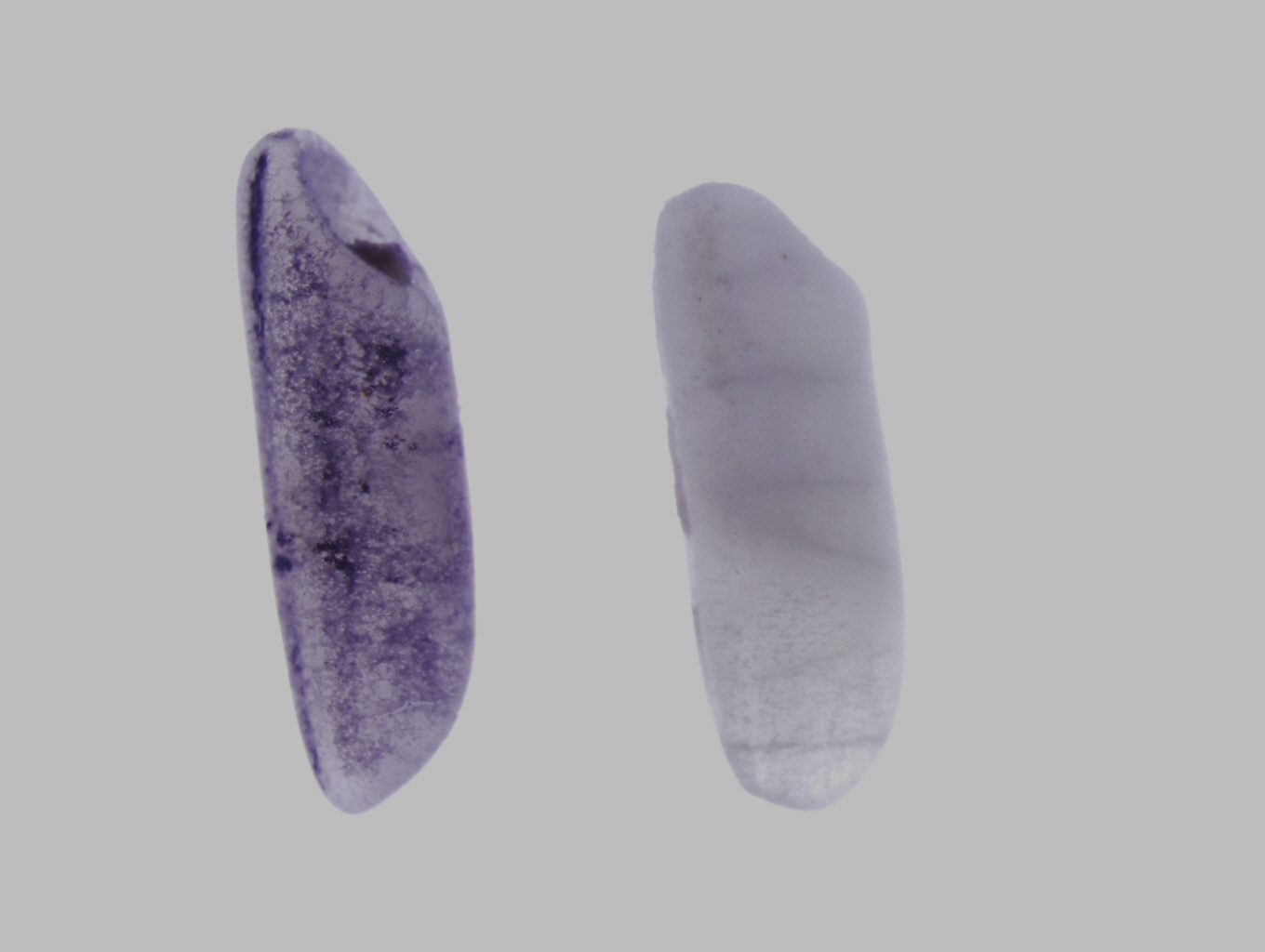


a

b

c

d

Transit peptide

α- helix

β- helix

C- helix

D- helix

F- helix

S**upplementary Fig. 5  *In silico* analysis o f amino acid sequence of ferritin from soybean and rice**
